# Pregnane X Receptor (PXR) Polymorphisms and Cancer Treatment

**DOI:** 10.3390/biom11081142

**Published:** 2021-08-02

**Authors:** Aikaterini Skandalaki, Panagiotis Sarantis, Stamatios Theocharis

**Affiliations:** First Department of Pathology, Medical School, National and Kapodistrian University of Athens, 11527 Athens, Greece; katerinaskan@med.uoa.gr (A.S.); psarantis@med.uoa.gr (P.S.)

**Keywords:** PXR, polymorphisms, SNP, cancer, treatment, pharmacogenomics

## Abstract

Pregnane X Receptor (PXR) belongs to the nuclear receptors’ superfamily and mainly functions as a xenobiotic sensor activated by a variety of ligands. PXR is widely expressed in normal and malignant tissues. Drug metabolizing enzymes and transporters are also under PXR’s regulation. Antineoplastic agents are of particular interest since cancer patients are characterized by significant intra-variability to treatment response and severe toxicities. Various PXR polymorphisms may alter the function of the protein and are linked with significant effects on the pharmacokinetics of chemotherapeutic agents and clinical outcome variability. The purpose of this review is to summarize the roles of PXR polymorphisms in the metabolism and pharmacokinetics of chemotherapeutic drugs. It is also expected that this review will highlight the importance of PXR polymorphisms in selection of chemotherapy, prediction of adverse effects and personalized medicine.

## 1. Introduction

Pregnane X receptor (PXR), also known as nuclear receptor subfamily 1 group I member 2 (NR1I2) and steroid and xenobiotic receptor (SXR), is an orphan receptor of the nuclear receptor gene superfamily and plays a key role in the metabolism of xenobiotics and endobiotics [1,2,3,4,5,6]. Human PXR (hPXR), a 49.7 kDa protein of 434 amino acids, is the product of the NR1I2 gene which is located in chromosome 3 (3q12-q13.3) and consists of approximately 40 kb [7,8]. hPXR is mostly expressed in normal liver tissue, the small intestine and the kidney whereas the PXR expression in tissues like stomach, ovaries, lungs, breast and peripheral blood cells is less frequent [9,10]. PXR expression in neoplastic tissues has also been reported, which differs from the expression levels in normal tissues [6,11].

**Figure 1:**
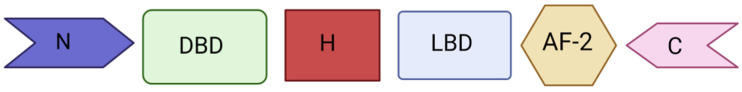
Structure of PXR protein. PXR consists of a ligand-depended activation function 2 (AF-2) and a highly conserved DNA-binding domain (DBD) in the N-terminal with two zinc-fingers while the DBD is connected to the C-terminal ligand binding domain (LBD) using a hinge.

The structure of the PXR protein is presented in Figure 1. The enlarged, hydrophobic pocket of PXR enables it to accommodate a larger and more diverse number of ligands than the rest of the nuclear receptors, such as endobiotics, pharmaceutical and herbal compounds, environmental factors and dietary supplements and other xenobiotics [12,13].

## 2. PXR Function

**Figure 2:**
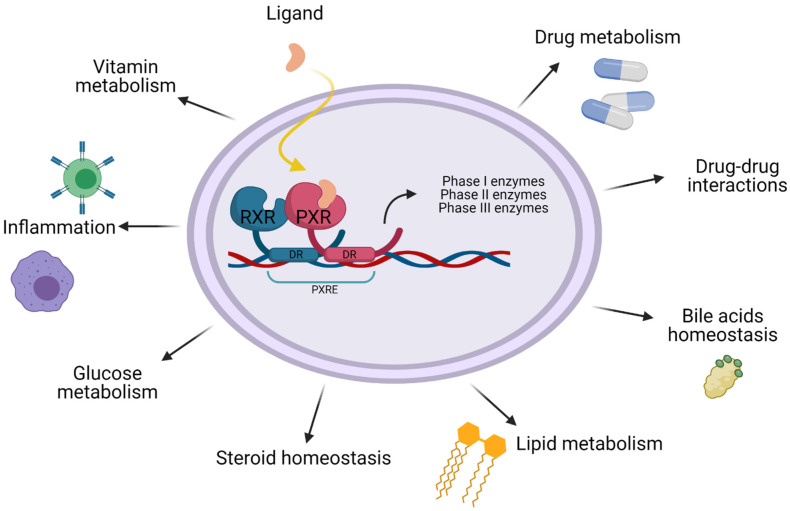
Functions of PXR. Besides being a significant xenobiotic regulator PXR also seems to have an important role in lipid and glucose metabolism, bile acid detoxification, steroid homeostasis, inflammation and vitamin metabolism.

As a master regulator of xenobiotic response PXR adjusts the expression of many phase I and phase II drug metabolizing enzymes (DME), such as cytochrome P450, uridine diphosphate (UDP)-glucuronosyltransferases, sulfotransferases and carboxylesterases [13,14]. PXR also regulates drug-efflux pumps multi-drug resistance gene 1 (MDR1), MDR2, ATP-binding cassette transporter C 2 (ABCC) and anion-transporting polypeptide 2 (OATP) [14,15,16]. All the aforementioned enzymes, under the control of PXR play a significant role in the biotransformation, metabolism and clearance of therapeutic agents, including chemotherapeutic agents, that may result in drug-drug, drug-herb and drug-food interactions, toxicity, adverse effects and reduced efficacy [13,14,16,17]. The overall functions of PXR protein are presented in Figure 2.

It has also been reported PXR’s participation in tumor cell proliferation and growth, apoptosis and metastasis as well as in liver regeneration and hepatic proliferation, indicating the important role of PXR in cancer [14,18].

Various nonsynonymous polymorphisms have been identified in essential domains of NR1I2, including DBD and LBD affecting either the DNA or the ligand binding process, while mutations in the 5′ UTR could affect PXR expression, resulting in modifications in drug metabolism and pharmacokinetics as well as contributing to drug resistance [3,12,19]. Polymorphisms located in gene exons can modify the LBD or the DBD domains of PXR, changing the interactions between these domains and ligands, gene promoters and co-regulators, while polymorphisms located in non-coding regions can affect the regulation of transcription and translation [3,20]. In the 3′ UTR of NR1I2 gene, specifically in the miRNA target sequence, several single nucleotide polymorphisms (SNPs) have been described to alter post-transcriptional micro-RNA (mi-RNA)-mediated regulation of PXR expression, creating or deleting regions of interaction with miRNAs, as *NR1I2* rs1054190 (C > T) and *NR1I2* rs1054191 (G > A), respectively [21]. Studies have also identified NR1I2 SNPs in exons, which initiate drastic changes in PXR in vivo but with very low frequencies in the population thus requiring very large cohort studies of patients [20].

## 3. Regulation

When PXR is not linked with an agonist its action is regulated by transcriptional co-repressors like nuclear receptor co-repressor 1 (NCoR1) and NCoR2 which inhibit PXR’s transcription via histone deacetylases (HDACs) activity [11,18]. When a ligand binds to PXR, through the LBD, the receptor heterodimerizes with retinoid X receptor (RXR), binds to xenobiotic response elements (XREs) and hormone response elements (HREs) and changes the status of coregulators (like co-repressors and co-activators, such as steroid receptor coactivator 1 and 3 [SRC-1 and SRC-3]), which remodel chromatin via histone acetyltransferase (HAT) activity and regulate transcription [11,22,23,24,25]. Once the ligand binds to the LBD, the AF-2 region at the C-terminus binds to specific amino-acid motifs of transcriptional coactivators and corepressors, resulting in the correct arrangement of the ligand in the PXR ligand-binding pocket [26,27,28]. PXR binds to different DNA response elements, including direct repeats (DRs) DR-4, DR-5 and everted repeats (ERs) ER-6 and ER-8 among others, while the receptor seems to have a higher binding preference for DNA-binding motif of DR-(5n + 4) [28,29]. Activated Nuclear Factor-kappa B (NF-κB) is reported to inhibit the activation and function of PXR, while inhibited NF-κB increases PXR activity and transcription of target genes. This mutual negative crosstalk between PXR and NF-κB indicated that PXR acts as a negative mediator of inflammation and immunity [30].

## 4. Post-Translational and Post-Transcriptional PXR Modifications

**Figure 3:**
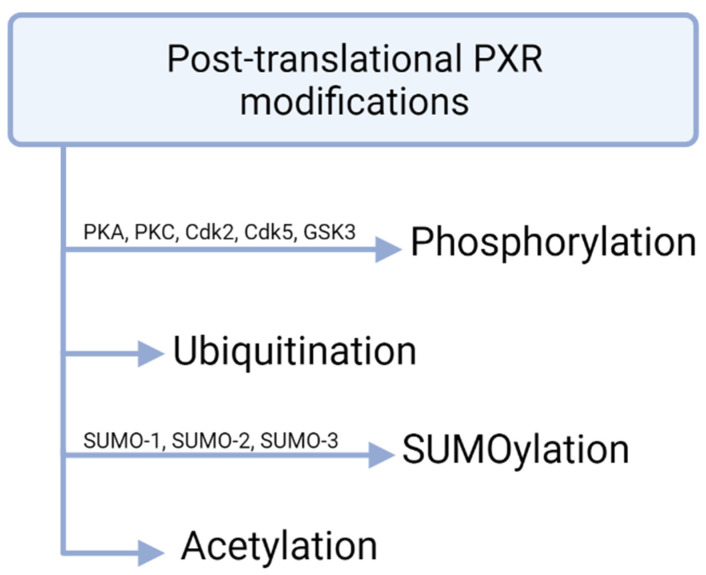
PXR modifications. Phosphorylation of PXR mostly reduces transcriptional activity. Ubiquitination is mediated by the 26S proteasome and SUMOylation takes place in lysine residues. Post-translational acetylation of PXR has been shown in vivo.

Apart from the direct ligand-depended activation PXR is subjected to post-translational modifications (PTMs) resulting in variations of PXR transcriptional activities which are presented in Figure 3 [31].

There is evidence that PXR acetylation interacts with PXR SUMOylation and may be mutually excluded, but further research is needed [17,32].

Micro-RNAs (mi-RNA) are reported to alter PXR expression via post-transcriptional modifications usually targeting the 3′-untraslated region (UTR) sequence of human PXR transcripts [33]. miR-30c-1-3p is identified as a PXR silencer, thus decreases the mRNA levels of PXR and alters CYP3A4 expression [33]. Other post-translational PXR regulators are miR-18a-5p, miR-148a, miR-34a, miR-150, miR-27a and miR-140-3p, which negatively regulate PXR expression, resulting in decreased CYP3A4 expression [34,35,36,37,38]. miR-449a is described to inhibit PXR through HNF4-a in human hepatocytes [39].

## 5. Agonists and Antagonists

PXR can bind to a variety of agonists due to the receptor’s large cavity, as mentioned above. Some endobiotic PXR ligands are bile acids and their precursors, progesterone, pregnenolone, 17-hydroxypregnenolone, cholesterol and it’s metabolites and lithocholic acid [12,14,40]. Regarding xenobiotic PXR ligands, these may include prescription drugs and anticancer agents like ritonavir, rifampicin, clotrimazole, cyclosporin, paclitaxel, Taxol, tamoxifen, dexamethasone, troglitazone, statins, nifedipine, spironolactone, endocrine disruptors and phenobarbital [12,40]. Other xenobiotic ligands are carotenoids, vitamins like vitamin K2 and vitamin E, herbal medicines like St. John’s Wort, Kava Kava, Gugulipid, Sweet Wormwood Herb, Schisandra and environmental pollutants such as organochlorine pesticides and polybrominated diphenyl ether flame retardants [12,41].

In order to prevent PXR-mediated drug-drug interactions and restrict the variability of efficacy of therapeutics, many PXR antagonists have been identified or been developed. In 2001 the first PXR antagonist was reported, ET-743, followed by numerous other compounds like ketoconazole, fluconazole, enilconazole, camptothecin, metformin, sulforaphane, sesamin, coumestrol, allyl isothiocyanate, algal carotenoid fucoxanthin, silybin and isosilybin [42,43,44]. Environmental toxins, such as polychlorinated biphenyls (PCBs) exhibit antagonistic activity, while highly chlorinated PCBs selectively antagonizing mPXR but not human PXR [45]. However, many of these compounds bind other targets at concentrations below the range that affects PXR, resulting in incapability to inhibit PXR in vivo [43]. Some of these antagonists, ketoconazole, coumestrol and metformin, are reported to inhibit PXR’s transactivation either via interfering with PXR’s coactivators or via binding in the AF-2 domain independently of PXR LBD, while ketoconazole is reported to be able to bind to two distinct PXR binding pockets either causing allosteric or direct inhibition of coactivator binding [42,43]. Ochatoxin A, a mycotoxin, has also been shown to significantly downregulate PXR activity in human primary hepatocytes [46,47]. Other antagonists are clotrimazole, dabrafenib, SR12813, Nelfinavir and SPA70 [48,49]. SPA70 interacts with hPXR LBD, is highly specific for hPXR and has selective downregulating effects [47,50,51,52]. Although several analogs of PXR antagonists have been synthesized many function as agonists due to the flexible and large cavity of PXR, which adapts to the shape of the ligands [43].

## 6. PXR and Cancer

As stated, PXR regulates the expression of a variety of target genes and can participate in many physiological and pathological conditions through complex cellular circuits as PXR manipulates the expression of target genes participating in biotransformation, inflammation, cell-cycle regulation, apoptosis, tissue growth and oxidative stress [47,53]. These biological functions of PXR have an impact on cancer initiation, promotion and progression, and on the outcome of chemotherapeutic agents, as PXR and its target genes are linked with multidrug resistance, poor chemotherapy outcome as well as detoxification, defense and homeostasis maintenance, which inhibit cancer development, highlighting PXR as a central target of cancer regulation [47]. PXR is described to be associated with various cancers, including breast, esophageal, prostate, ovaries, cervix, endometrial tissues, colon, pancreas, liver, lung and hematological malignancies [18,53,54,55]. Also PXR overexpression and altered subcellular location, due to mutation, is linked with endometrial, breast and colorectal cancer [16]. Esophageal adenocarcinoma and Barrett’s epithelium were associated with increased PXR mRNA levels, while the PXR protein was not detected in normal esophageal epithelium and was detected in the nuclei of cancer cells [54].

Due to the involvement of PXR in drug transporters’ and the gene expression and regulation of drug metabolizing enzymes, such as CYP3A4, which is responsible for metabolizing more than 50% of drugs that include chemotherapeutics, PXR significantly contributes to chemotherapy resistance and variations in the chemotherapeutic outcome [16,56]. PXR contributes to the chemotherapy outcome by interfering with the metabolism, drug resistance, tumor sensitivity, apoptosis and pharmacokinetics parameters of many chemotherapeutic agents, such as tamoxifen, irinotecan, vinblastine, doxorubicin, paclitaxel, cisplatin and ixabepilone in cancer cell lines and patients [11,14]. Several studies have indicated PXR involvement in tumor sensitivity to anticancer agents. Enhanced PXR activation via miRNA-30c repression by factor that binds to the inducer of short transcripts-1 (FBI-1) was linked with chemotherapeutic resistance in triple-negative breast cancer cell lines [57]. Dabrafenib-induced hPXR activation in colon cancer cell lines was associated with enhanced expression of PXR target genes, including CYP3A4 and CYP2D6. This study indicates the potential impact of dabrafenib on its own metabolism or the metabolism of other therapeutic agents combined with dabrafenib via PXR regulation [58].

Considering that cancer patients are treated with multidrug regimens, PXR-mediated drug-drug interactions and drug toxicity are very important, as is PXR-mediated chemoresistance, which affects clinical outcome. So far, the exact molecular mechanisms are unclear, but SNPs within the NR1I2 gene could be a possible mechanism involved in MDR and altered clinical outcome of antineoplastic agents [56]. Up to now numerous SNPs have been reported in the NR1I2 gene, some of which may have an impact on the course of cancer treatment. In view of the above considerations, the aim of this review is to highlight the significance of researched SNPs in efficacy and toxicity of antineoplastic agents.

## 7. PXR Polymorphisms and Cancer Pharmacogenetics

### 7.1. Gastrointestinal Cancer

Pharmacogenomics have recently widely entered the personalization of CRC treatment, specifically centering on the genetic variability in metabolism-related genes, such as NR1I2. In Caucasian patients with metastatic colorectal cancer (CRC) treated with FOLFIRI (irinotecan, bolus and continuous-infusion fluorouracil, leucovorin), the T allele at NR1I2 rs1054190 (C > T) was associated with worse overall survival (OS) and progression-free survival (PFS), identifying NR1I2-rs1054190 polymorphism as a potential prognostic marker of OS [59]. The aforementioned SNP, located in 3′UTR, has been linked with downregulation of PXR and regulation of the expression of PXR via miRNA mechanisms [16,56,58]. Patients with metastatic CRC treated with FOLFIRI or FOLFIRINOX carrying the A allele of NR1I2 rs10934498 (G > A, G > C, G > T) were associated with a decreased area under the curve (AUC) of SN-38, the active metabolite of irinotecan, decreased biliary index and a decreased risk of grade 3–4 hematotoxicity. This study also highlighted the association between patients carrying the T allele at NR1I2 rs3814055 (C > T) as well as patients carrying the C allele at NR1I2 rs1523127 (C > A) and increased risk of grade 3–4 hematotoxicity, while patients with the G allele at NR1I2 rs2472677 (C > G, C > T) exhibited higher risk for all types of grade 3–4 toxicity [60]. Asian patients with gastrointestinal stromal tumors (GISTs) carrying the T allele at NR1L2 rs3814055 (C > T) exhibited lower steady-state imatinib dose-adjusted plasma concentrations than patients with the wild type (CC), while in the same study patients carrying the mutant T allele at NR1L2 rs3814055 (C > T) showed significantly lower incidence rate of continuous edema, an adverse effect of imatinib [61]. These results are in agreement with the results from Yi Qian et al. where patients with the TT genotype of NR1I2 rs3814055 (C > T) were described to have lower unbound imatinib dose-adjusted concentration [62].

### 7.2. Breast Cancer

Drug resistance to chemotherapeutic agents and toxicity are often noticed in breast cancer patients during treatment and worsen the chemotherapy outcome [16,63]. In Caucasian women with breast cancer treated with FAC (doxorubicin, 5′-fluorouracil and cyclophosphamide) the presence of NR1L2 rs3732359 (G > A) was an independent predictor of OS as patients carrying the AA genotype were described to have a 2 times higher risk of death compared to homozygous for the wild type allele (GG) and heterozygous (AG) [63]. Regarding the clearance of doxorubicin in Asian women with invasive breast cancer treated with adjuvant chemotherapy with doxorubicin/cyclophosphamide, patients carrying the haplotype cluster tagged by IVS6-17C > T (NR1I2 rs2276707) and 2654T > C (NR1I2 rs3814058) were characterized by reduced doxorubicin clearance [7]. SNPs NR1I2 rs3732360 (C > T, C > G), rs1054190 (C > T) and rs1054191 (G > A) are described to be linked with change in doxorubicin pharmacokinetics via altering the miRNA mediated post-transcriptional regulation of PXR in Asian (Indian) breast cancer patients [16]. Haplotype PXR*1B, which consists of NR1I2 rs2276707 (C > G, C > T) and NR1I2 rs3814058 (T > C), has been linked with decreased plasma expression of PXR in hepatic tissue, while NR1I2 rs3732359 and NR1I2 rs3732360, located in the 3′UTR, affect the effectiveness of miRNA and PXR mRNA resulting in modifications of PXR’s expression [7,16].

### 7.3. Renal Cell Carcinoma

Sunitinib is currently registered as first-line and second-line therapy for metastatic renal cell carcinoma (mRCC) and its efficacy may be dependent on its exposure, regulated by efflux pumps and metabolizing enzymes. Clear-cell RCC patients, treated with sunitinib, with the T allele at NR1I2 rs2276707 (C > T, C > G) and patients carrying the T allele at NR1I2 rs3814055 (C > T) were described to have a shorter PFS and a shorter OS for the T allele at rs3814055 (C > T), results confirmed by Beuselinck, B. et al. [64,65]. Another study described the link between the response rate (RR) for pazopanib in RCC patients carrying the NR1I2 rs3814055 (C > T). Patients carrying the T allele showed significantly reduced RR with a potential consequence for drug exposure and a trend to have reduced PFS compared with carriers of the wild type genotype (CC) [66,67].

### 7.4. Others

The presence of NR1I2 rs6785049 (G > A, G > T) or rs3814055 (C > T) was linked with inter-patient variability of temsirolimus pharmacokinetics and toxicity in patients with metastatic bladder cancer. Patients with the T allele of NR1L2 rs3814055 or the G allele of NR1L2 rs6785049 showed significantly lower frequency of adverse events, while patients homozygous for the NR1L2 rs3814055 wild type C allele (CC) and patients homozygous for the NR1L2 rs6785049 mutant A allele (AA) exhibited higher frequencies of severe temsirolimus toxicity. This study also indicated that NR1L2 rs6785049 GG genotype was correlated with increased exposure to active entities (AUCsum) and that NR1L2 rs3814055 TT genotype was linked with extended tesmirolimus T1/2 although the effect was not additive [68].

Patients with nasopharyngeal cancer treated with docetaxel, carrying the mutant allele of SNPs NR1L2 rs3732359 (G > A), rs3732360 (C > T, C > G) or rs3814058 (T > C), exhibited a decrease in nadir hemoglobin from baseline in cycle 1 but did not show any correlation with the pharmacokinetics of docetaxel [69]. Also, homozygous for the mutant allele and heterozygous (CC + TC) Asian non-small cell lung cancer (NSCLC) patients treated with platinum-based chemotherapy with NR1I2 rs3814058 (T > C) exhibited higher risk of hematological toxicity than patients homozygous for the wild type allele (TT) [70]. Asian patients with chronic myeloid leukemia (CML) treated with bosutinib, who also carried the genotypes (GG) or (TT) of NR1L2 rs6785049 (G > A, G > T) or NR1L2 rs2276707 (C > T), respectively, exhibited lower bosutinib through plasma concentration (C0) than patients carrying alleles A of rs6785049 or allele C of rs2276707, while patients carrying both (GG) and (TT) genotypes showed lower bosutinib C0 than other genotypes, indicating increased clearance of the anticancer agent [71].

**Figure 4:**
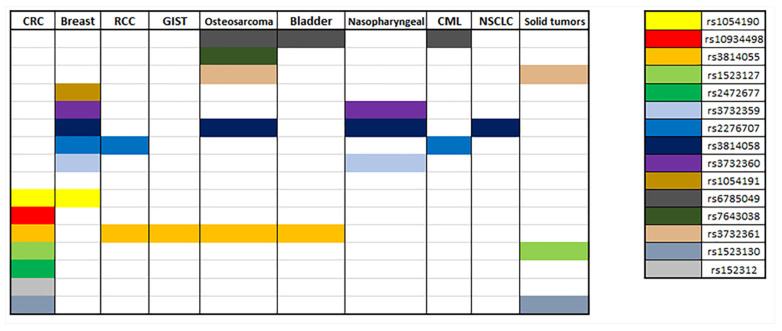
Summary of correlations of NR1I2 polymorphisms with different types of cancer.

In docetaxel-based treatment for patients with solid tumors the SNP NR1I2 rs3732359 (G > A) was significantly associated with docetaxel-induced myelosuppression grade ≥3, as carriers of the wild type G allele were of higher myelosuppression risk [72]. Another study indicated that, in cancer patients who received carboplatin plus paclitaxel as chemotherapy, the NR1I2 rs1523130 (T > A, T > C, T > G) and rs1523127 (T > G) were related, with altered sensitivity to thrombocytopenia; as the A allele of rs1523130 and the G allele of rs1523127 exhibited a recessive and genotypic effect, the AA genotype of rs15233130 and the GG genotype of rs1523127 were correlated with a decreased sensitivity to thrombocytopenia. This study also showed that carriers of two copies of the ATG haplotypes of NR1I2 rs1523130 (T > A, T > C, T > G), NR1I2 rs1523127 (T > G) and NR1I2 rs3814055 (C > T) were less sensitive to thrombocytopenia [73]. Osteosarcoma patients treated with methotrexate (MTX) presented differences in MTX pharmacokinetics and toxicities depending on their genotype. Patients carrying the SNPs NR1I2 rs3814055 (C > T) and rs7643038 (A > G) exhibited longer first half-life of MTX, while SNPs NR1I2 rs6785049 (G > A, G > T) and rs3732361 (G > A) were linked with higher 48 h MTX concentration. The same study indicates correlation between SNPs NR1I2 rs3732361, rs3814058 and rs6785049 and lower risk of hepatic and bone marrow toxicity [74]. All of the above are summarized in Figure 4 and Table 1.

## 8. Discussion

Numerous NR1I2 SNPs have been researched regarding their association with pharmacotherapy outcome and severe toxicities. In Chinese Han tuberculosis patients who received anti-tuberculosis treatment, with NR1I2 rs7643645 (A > G) were associated with increased risk of anti-tuberculosis drug-induced hepatotoxicity (ATDH) (GG genotype), while carriers of the NR1I2 rs2276707 (C > T) were linked with reduced risk of ATDH [75]. These results were at conflict with the results from Yu Wang et al., where the G allele of NR1I2 rs7643645 was associated with reduced risk of ATDH [76]. NR1I2 polymorphisms detected in patients who received cyclosporin (CsA) after their first renal transplantation were correlated with altered CsA C0/D and C2/D after the first month of transplantation, as the haplotype cluster PXR*1B tagged by NR1I2 rs2276707 and rs3814058 was correlated with increased CsA C2/D [77]. A study also indicated the importance of NR1I2 rs13059232 (T > C) as a biomarker for clopidogrel therapy in acute ischemic stroke (IS) patients, since patients carrying the CC genotype exhibited poorer clinical outcome than patients carrying the T allele, while comparable results were not observed in the aspirin cohort [78]. HIV-positive patients treated with atazanavir and ritonavir and carrying the G allele of NR1I2 rs1523130 (T > G) exhibited higher ritonavir intracellular concentrations [79]. Furthermore, HIV-positive patients, homozygous for the mutant allele of NR1I2 rs2472677 (C > T) and treated with atazanavir, were associated with a 17.0% higher clearance of atazanavir [80]. Alzheimer’s patients treated with memantine and carrying the T allele of NR1I2 rs1523130 (T > C) were reported to exhibit slower memantine clearance [81]. It has also been reported that the Cssmin of voriconazole, given to patients with hematological malignancies, is affected significantly by SNPs NR1I2 rs2461817 (A > C), rs7643645 (A > G), rs3732359 (G > A), rs3814057 (A > C) and rs6785049 (G > A) [82]. The studies above highlight the possible significant involvement of PXR in the pharmacokinetics of therapeutic agents, the therapeutic outcome along with adverse effects and severe toxicity. Especially in neoplastic diseases, where patients exhibit important intra-individual variations of the therapeutic outcome and severe toxicities, identification and characterization of PXR polymorphisms are deemed necessary.

Although chemotherapy is still the main strategy followed for the treatment of many solid tumors and systematic malignancies, it is characterized by significant inter-individual heterogeneity of chemotherapeutic response, toxicity and drug resistance, which alter the clinical outcome of the chemotherapeutic treatment. Evidently, better understanding of factors that determine the chemotherapeutic response will help to detect patients who are at risk of displaying severe toxicities or benefit the most from a specific therapeutic combination, providing personalized treatment [14,83]. To achieve this goal extended research which incorporate modern techniques like next generation sequencing (NGS) is required as well as conducting more clinical studies with a larger cohort of patients.

## 9. Conclusions

PXR is characterized as a master regulator of xenobiotic and endobiotic metabolism, since it modulates the expression of significant enzymes involved in drug distribution, metabolism and clearance, and has been linked with various functions, such as inflammation, drug-drug interactions, detoxification, and vitamin and bile acid metabolism. PXR polymorphisms in the NR1I2 gene are considered to have noteworthy consequences on the protein’s function, such as abnormal DNA binding and changes in target genes transactivation and expression; thus detection and identification of functional PXR SNPs, and their distribution in the different populations and ethnicities, are crucial for understanding and explaining the mechanisms behind the variations in drug pharmacokinetics and clinical outcome. Cancer patients receiving chemotherapeutic agents are often characterized by variability in chemotherapeutic outcome, severe adverse events, resistance to therapy and drug-drug interactions due to polypharmacy. Since PXR regulates the metabolism and pharmacokinetics of the majority of the antineoplastic agents, it is important to study the effects PXR polymorphisms have on them so we can enhance the personalized therapy of cancer therapy. The current review highlights the importance of PXR polymorphisms in cancer precision medicine. Current literature data report that gene PXR polymorphisms appear to interfere with pharmacokinetics, metabolism and toxicity of antineoplastic factors. Some of these seem to affect the response and toxicity of more than one antineoplastic therapy, for instance *NR1I2* rs3814055 has been linked with variability in pharmacokinetics parameters and toxicity of FOLFIRINOX/FOLFIRI, imatinib, sunitinib, pazopanib, methotrexate and temsirolimus. The study of PXR SNPs in combination with the development of PXR antagonists with antineoplastic therapies that activate PXR receptors have the potential to decrease or eradicate adverse events, toxicity and chemoresistance, enhancing precision medicine in cancer. In summary, 15 polymorphisms were found to be associated with variations in chemotherapy clinical outcome or toxicity in cancers such as breast, gastrointestinal and renal, all of them located in non-translated regions of the NR1I2 gene. Further information is required to fully understand the role of PXR SNPs in response to treatment and clinical outcome of cancer patients, although the currently available data indicate the significance of PXR polymorphisms for the pharmacotherapy of cancer patients.

## Figures and Tables

**Table 1:** PXR polymorphisms and their associations with efficacy, toxicity and pharmacokinetics of chemotherapeutic agents.

SNP (rs)	Localization	Pathology	Therapy	Patients	Association	Ref.
rs1054190 (C > T)	3′-UTR	mCRC	FOLFIRI	247 Italian (discovery cohort) 90 Canadian (Replication cohort)	Worse OS and PFS (T allele)	[59]
		Breast Cancer	Doxorubicin	96 South Indian	Change in doxorubicin pharmacokinetics via miRNA altering	[16]
rs10934498 (G > A, G > C, G > T)	Intron 1	mCRC	FOLFIRI/ FOLFIRINOX	109 French	Decreased AUC of SN-38 Decreased biliary index Decreased risk of grade 3–4 hematotoxicity (A allele)	[60]
rs3814055 (C > T)	5′-UTR	mCRC	FOLFIRI/ FOLFIRINOX	109 French	Increased risk of grade 3–4 hematotoxicity (T allele)	[60]
		GISTs	Imatinib	68 Asian 62 patients	Decreased imatinib plasma concentrations and lower edema incidence (T allele)	[61,62]
		RCC	Sunitinib	136 patients	Shorter PFS, OS (T allele)	[64,65]
		RCC	Pazopanib	397 patients	Reduced RR, PFS, OS (T allele)	[66,67]
		Bladder cancer	Temsirolimus	54 patients	Decreased frequency of adverse events (T allele) High frequency of severe toxicity (CC genotype) Extended temsirolimus T1/2 (TT genotype)	[68]
		Osteosarcoma	MTX	59 patients	Increased first T1/2 of MTX (T allele)	[74]
rs1523127 (C > A)	5′-UTR	mCRC	FOLFIRI/ FOLFIRINOX	109 French	Increased risk of grade 3–4 hematotoxicity (C allele)	[60]
rs2472677 (C > G, C > T)	Intron 2	mCRC	FOLFIRI/ FOLFIRINOX	109 French	Increased risk of all type of grade 3–4 toxicity (G allele)	[60]
rs3732359 (G > A)	3′-UTR	Breast cancer	FAC	305 Caucasian	Increased OS (G allele)	[63]
		Nasopharyngeal cancer	Docetaxel	50 Asian	Decrease in nadir hemoglobin from baseline (G allele)	[69]
		Solid tumors	Docetaxel	110 Asian	Docetaxel-induced myelosuppresion grade ≥3 (G allele)	[72]
rs2276707 (C > T, C > G)	Intron 7	Breast cancer	Doxorubicin/ Cyclophosphamide	62 Asian	Haplotype cluster (rs2276707 and rs3814058) associated with reduced doxorubicin clearance	[7]
		RCC	Sunitinib	136 patients	Shorter PFS, OS (T allele)	[64,65]
		CML	Bosutinib	30 Asian	Increased bosutinib clearance (TT genotype)	[71]
rs3814058 (T > C)	3′-UTR	Breast cancer	Doxorubicin/ Cyclophosphamide	62 Asian	Haplotype cluster (rs2276707 and rs3814058) associated with reduced doxorubicin clearance	[7]
		Nasopharyngeal cancer	Docetaxel	50 Asian	Decrease in nadir hemoglobin from baseline (C allele)	[69]
		NSCLC	Platinum-based	262 Asian	High risk of hematological toxicity (C allele)	[70]
		Osteosarcoma	MTX	59 patients	Reduced risk of hepatotoxicity/bone marrow toxicity (C allele)	[74]
rs3732360 (C > T, C > G)	3′-UTR	Breast cancer	Doxorubicin	96 South Indian	Change in doxorubicin pharmacokinetics via miRNA altering	[16]
		Nasopharyngeal cancer	Docetaxel	50 Asian	Decrease in nadir hemoglobin from baseline (T allele)	[69]
rs1054191 (G > A)	3′-UTR	Breast cancer	Doxorubicin	96 South Indian	Change in doxorubicin pharmacokinetics via miRNA altering	[16]
rs6785049 (G > A, G > T)	Intron 6	Bladder cancer	Temsirolimus	54 patients	Decreased frequency of adverse events (G allele) Increased exposure to active entities (GG genotype) High frequency of severe toxicity (AA genotype)	[68]
		CML	Bosutinib	30 Asian	Increased bosutinib clearance (GG genotype)	[71]
		Osteosarcoma	MTX	59 patients	Increased 48-h MTX concentration (G allele) Reduced risk of hepatotoxicity/bone marrow toxicity (G allele)	[74]
rs7643038 (A > G)	5′-UTR	Osteosarcoma	MTX	59 patients	Increased first T1/2 of MTX (G allele)	[74]
rs3732361 (A > G, A > C)	3′-UTR	Osteosarcoma	MTX	59 patients	Increased 48-h MTX concentration (G allele) Reduced risk of hepatotoxicity/bone marrow toxicity (G allele)	[74]
rs1523130 (T > A, T > C, T > G)	5′-UTR	mCRC	Irinotecan	109 Caucasian	Reduced APC and NPC metabolism (T allele)	[60]
		Solid tumors	Carboplatin/Paclitaxel	201 patients	Reduced sensitivity to thrombocytopenia (AA genotype) ATG haplotype (rs1523130, rs3814055, rs1523127) linked to reduced sensitivity to thrombocytopenia	[73]
rs152312 (C > T)	5′-UTR	mCRC	Irinotecan	109 Caucasian	Reduced NPC metabolism (C allele)	[60]

## Data Availability

Data sharing not applicable. No new data were created or analyzed in this study.
